# Repression of anti-proliferative factor *Tob1 *in osteoarthritic cartilage

**DOI:** 10.1186/ar1479

**Published:** 2005-01-11

**Authors:** Mathias Gebauer, Joachim Saas, Jochen Haag, Uwe Dietz, Masaharu Takigawa, Eckart Bartnik, Thomas Aigner

**Affiliations:** 1 Aventis Pharma Deutschland, Functional Genomics, Sanofi-Aventis, Frankfurt, Germany; 2 Sanofi-Aventis, Disease Group Thrombotic Diseases/Degenerative Joint Diseases, Frankfurt, Germany; 3 Osteoarticular and Arthritis Research, Department of Pathology, University of Erlangen-Nürnberg, Germany; 4 Department of Biochemistry and Molecular Dentistry, Okayama University Graduate School of Medicine and Dentistry, Okayama, Japan

**Keywords:** bone morphogenetic protein, cartilage, chondrocytes, gene expression, proliferation

## Abstract

Osteoarthritis is the most common degenerative disorder of the modern world. However, many basic cellular features and molecular processes of the disease are poorly understood. In the present study we used oligonucleotide-based microarray analysis of genes of known or assumed relevance to the cellular phenotype to screen for relevant differences in gene expression between normal and osteoarthritic chondrocytes. Custom made oligonucleotide DNA arrays were used to screen for differentially expressed genes in normal (*n *= 9) and osteoarthritic (*n *= 10) cartilage samples. Real-time polymerase chain reaction (PCR) with gene-specific primers was used for quantification. Primary human adult articular chondrocytes and chondrosarcoma cell line HCS-2/8 were used to study changes in gene expression levels after stimulation with interleukin-1β and bone morphogenetic protein, as well as the dependence on cell differentiation. *In situ *hybridization with a gene-specific probe was applied to detect mRNA expression levels in fetal growth plate cartilage. Overall, more than 200 significantly regulated genes were detected between normal and osteoarthritic cartilage (*P *< 0.01). One of the significantly repressed genes, *Tob1*, encodes a protein belonging to a family involved in silencing cells in terms of proliferation and functional activity. The repression of *Tob1 *was confirmed by quantitative PCR and correlated to markers of chondrocyte activity and proliferation *in vivo*. *Tob1 *expression was also detected at a decreased level in isolated chondrocytes and in the chondrosarcoma cell line HCS-2/8. Again, in these cells it was negatively correlated with proliferative activity and positively with cellular differentiation. Altogether, the downregulation of the expression of *Tob1 *in osteoarthritic chondrocytes might be an important aspect of the cellular processes taking place during osteoarthritic cartilage degeneration. Activation, the reinitiation of proliferative activity and the loss of a stable phenotype are three major changes in osteoarthritic chondrocytes that are highly significantly correlated with the repression of *Tob1 *expression.

## Introduction

Osteoarthritis is the most common disabling condition of humans in the western world. Although osteoarthritis is mainly a disease and functional loss of the articular cartilage covering the joint surfaces, it is clearly the cells that are the active players during the disease process [1]. Whatever pleomorphisms the cellular reaction patterns display at first sight during the osteoarthritic disease process, they can be basically summarized in three categories (reviewed in [2]). First, the chondrocytes can degenerate or proliferate. Second, chondrocytes can activate or deactivate their synthetic anabolic or catabolic matrix-degrading activity by increasing or decreasing anabolic or catabolic gene expression. Last, chondrocytes can undergo phenotypic modulations implicating an overall severely altered gene expression profile of the cells in the diseased tissue. In fact, several distinct phenotypes of chondrocytes are known to occur *in vitro*, *in vivo *during fetal development and potentially also in the disease process itself, but new markers are required for the more accurate characterization of cellular behavior [3]. This will allow further analysis of the underlying pathology to develop therapeutic approaches that could delay, stop, or even reverse cartilage degeneration.

In many laboratories single and multiple gene analyses have been performed on normal and osteoarthritic cartilage specimens; however, a global overview of disease-associated changes is not available. This highlights the need for establishing a broader gene expression profile of osteoarthritic chondrocytes by modern screening technologies so as to characterize more properly the cellular events and regulatory pathways directly involved in cartilage destruction. In the present study, we designed a custom-made oligonucleotide-based microarray to screen for differentially expressed genes in normal and osteoarthritic cartilage specimens. We found that *Tob1*, a gene involved in cell cycle regulation and cell quiescence [4,5], was significantly repressed in osteoarthritic chondrocytes. This was confirmed by quantitative polymerase chain reaction (qPCR) and further analyzed in adult articular chondrocytes *in vitro *and *in vivo*.

## Materials and methods

### Donors for mRNA expression analysis

For the study of mRNA expression levels within the tissue, cartilage from human femoral condyles of normal knee joints was used. Normal articular cartilage (*n*_qPCR _= 10, age range 45–88 years, mean age 64.1 years; *n*_array _= 9, age range 37–83 years, mean age 59 years) was obtained from donors at autopsy, within 48 hours of death. Osteoarthritic cartilage samples from late-stage osteoarthritic joint disease were obtained from patients undergoing total knee replacement surgery (*n*_qPCR_= 15, age range 63–85 years, mean age 74.5 years; *n*_array _= 10, age range 57 to 84 years, mean age 76 years). The cartilage was frozen in liquid nitrogen immediately after removal and stored at -80°C until required for RNA isolation.

Cartilage was considered to be normal according to a macroscopic scoring system of the opened joint: this mainly included normal synovial membrane, normal synovial fluid, no significant overall softening or surface fibrillation (except on the tibial plateau, which is basically found in all specimens depending on age). The Mankin's grade of histological plugs taken was less than 3. Osteoarthritic cases fulfilled the criteria published by the American College of Rheumatology [6]. Cases of rheumatoid origin were excluded from the study.

### Isolation of primary human articular chondrocytes; stimulation with interleukin (IL)-1β and bone morphogenetic protein (BMP)-7

Normal human knee articular cartilage was obtained from six normal cases at autopsy within 48 hours of death. Cartilage pieces were finely chopped and chondrocytes were isolated enzymatically as described previously [7]. Chondrocytes were either plated in high-density monolayer cultures or cultured in alginate beads. Cultures were maintained for 48 hours in serum-free Dulbecco's modified Eagle's medium/F12 medium (Gibco BRL, Eggstein, Germany) supplemented with 1% penicillin/streptomycin solution (Gibco BRL) and 50 μg/ml ascorbate (Sigma, Taufkirchen, Germany) and 10% fetal calf serum (Biochrom, Berlin, Germany).

After 48 hours, primary (non-passaged) chondrocytes were stimulated with 1 ng/ml IL-1β (R&D System, Minneapolis, MN, USA) in DMEM/F12 medium, 100 ng/ml recombinant human BMP-7 (Stryker Biotech, Hopkinton, MA, USA) or cultivated in medium alone for 24 hours with no medium change afterwards. The same experiments were performed in parallel in the presence and in the absence of 10% fetal calf serum. At the end of the culturing/stimulation period the cells were washed in sterile phosphate-buffered saline (PBS), lysed in 350 μl of lysate RLT buffer/10^6 ^cells and stored at -80°C.

### Culture of HCS-2/8 cells

The human HCS-2/8 chondrosarcoma cell line (around passage 50–55) [8,9] was cultured in DMEM (PAA, Linz, Austria) supplemented with 20% fetal bovine serum (Gibco BRL) and with 50 μg/ml ascorbate (Sigma) in a humidified atmosphere of 5% CO_2 _at 37°C as described [9]. Cells were seeded at 10^5 ^cells/cm^2 ^and grown for 3 days to obtain subconfluent stage cultures, at 2 × 10^5^/cm^2 ^and cultured for 7 days to obtain confluent stage cultures, and at 6 × 10^5^/cm^2 ^and grown for 10 days for over-confluent stage cultures.

### RNA isolation from articular cartilage and isolated articular chondrocytes

Total RNA from both cartilage tissue and isolated chondrocytes was isolated as described previously [10,11]. The quality of total RNA samples was checked by agarose-gel electrophoresis and with the Bioanalyzer RNA 6000 Nano assay (Agilent, Waldbronn, Germany).

### Construction of the SensiChip cartilage microarray

The SensiChip technology is a two-color microarray platform using the Planar Wave Guide technology for microarray detection [12], which increases signal-to-noise ratios and thereby the sensitivity of hybridization experiments. The arrays were spotted in duplicate with 70-mer oligonucleotides representing the 3' untranslated region (UTR) of about 340 human cartilage-relevant genes, whereas one single gene was represented by one 70-mer oligonucleotide.

### Expression profiling with the SensiChip two-color DNA-microarray platform

Total RNA (250 ng) from osteoarthritic cartilage (10 samples) and pooled normal cartilage was amplified and labeled with Cy3-UTP and Cy5-UTP respectively (Amersham Pharmacia) using the MessageAmp aRNA kit (Ambion). After clean-up of the complementary RNA (cRNA) with the RNeasy kit (Qiagen), 5 μg of Cy3-labeled cRNA from osteoarthritic cartilage was mixed with 5 μg of Cy5-labeled cRNA from pooled normal cartilage. cRNA was fragmented by incubation with 40 mM Tris-acetate, pH 8.1, 100 mM potassium acetate, 30 mM magnesium acetate for 15 min at 95°C and desalted with a Microcon YM-10 concentrator (Millipore). Mixed Cy-dye labeled cRNA samples (600 ng) were hybridized for 16 hours on a SensiChip microarray (Qiagen) spotted in duplicate with 70-mer oligonucleotides representing the 3' UTR of selected genes. The gene-specific oligonucleotide sequences were designed by Operon by using GenBank accession numbers and proprietary algorithms. After washing steps performed in accordance with the manufacturer's standard protocol, arrays were scanned with the SensiChip Reader. The resulting array images were analyzed with SensiChip View 2.1 software (Qiagen) to quantify gene-specific signal intensities.

For quality control of RNA labeling and hybridization efficiency, oligonucleotides representing human housekeeping genes, negative and external bacterial spiking controls were also included. These sequences were prelabeled with fluorescent Cy3 and Cy5 dyes, and mixed in different concentrations into the hybridization solutions containing the labeled cRNA samples from human cartilage.

### Expression data analysis

All microarray scans were inspected visually and checked for quality on the basis of the performance of negative, housekeeping and externally added Cy3/Cy5-prelabeled spiking controls. Raw signal intensities from each scan were imported into the gene expression analysis software Resolver version 4.0 (Rosetta Biosoftware, Seattle, WA, USA). The software employs an error-modeling approach for the analysis of microarray data [13]. An error model specific for the SensiChip microarray platform was designed by Rosetta Biosoftware based on expression data from repeated hybridizations of the same RNA material to determine the variation of signal intensities. A complete description of the statistical methods used is available in the technology section of the Rosetta Biosoftware website .

All scans were pre-processed and normalized with the SensiChip error model to calculate *P *values and error bars for every gene expression profile. The *P *value represented the probability that an observed gene regulation was due to a measurement error. Gene regulation was considered as statistically significant if the calculated *P *value was below a threshold of 0.05. For normalization of expression data, the average brightness of the Cy3 and Cy5 channels respectively was used that was calculated from spots within a range from 30% to 85% of the signal intensity distribution of all spots. Scans from multiple experiments (replicates) were combined by averaging expression data with an error-weighted algorithm (also described in the statistical methods document available on the Rosetta Biosoftware website).

### Real-time quantitative PCR using TaqMan technology

Real-time PCR was used to detect human *Tob1*, *collagen type II*, *Ki-67*, *matrix metalloproteinase (MMP)-13 *and *glyceraldehyde-3-phosphate dehydrogenase *mRNA expression levels in human articular cartilage RNA samples. The primers (MWG Biotech, Ebersberg, Germany) and TaqMan probes (Eurogentec, Liège, Belgium) were designed using Primer Express™ software (Perkin Elmer). To be able to obtain quantifiable results for all genes, specific standard curves using sequence-specific control probes were performed in parallel to the analyses. Thus, for each gene a gene-specific cDNA fragment was amplified by the gene-specific primers (Table 1) and cloned into pGEM T Easy (Promega, Mannheim, Germany) or pCRII TOPO (Invitrogen, Karlsruhe, Germany). The cloned amplification product was sequenced to confirm correct cloning. Cloned standard probes were amplified with the plasmid amplification kit (Qiagen), linearized and used after careful estimation of the concentration (gel electrophoresis, photometry, and a fluorimetric assay for deoxyribonucleic acids (Picogreen; Molecular Probes, Eugene, OR, USA)). For the standard curves concentrations of 10, 100, 1000, 10,000, 100,000, and 1,000,000 molecules per assay were used (all in triplicate).

For the analyses of the different genes, a separate master mixture was made up for each of the primer pairs and contained a final concentration of 200 μM NTPs, 600 nM Roxbuffer and 100 nM TaqMan probe. For all genes the final reaction mixture contained, besides cDNA and 1 U polymerase (Eurogentec), forward and reverse primers, the corresponding probes, and MgCl_2 _at concentrations given in Table 1. All experiments were performed in triplicate.

### Immunofluorescence

Immunofluorescence studies were performed on paraformaldehyde-fixed paraffin-embedded specimens of normal (*n *= 5) and osteoarthritic (*n *= 5) articular cartilage. Sections were first incubated with the primary antibodies overnight, then with biotin-labeled goat anti-mouse antibodies (Dianova, Hamburg, Germany) and then with peroxidase-labeled streptavidin (Dianova). Subsequently, the tyramide amplification system (PerkinElmer, Boston, MA, USA) was used for signal amplification. Finally, the signals were detected with Cy5-labeled streptavidin (Dianova). Nuclear staining was again performed with 4,6-diamidino-2-phenylindole. The sections were evaluated by a (fluorescence) microscope (Olympus AX70) and photographed digitally.

To obtain optimal staining results various enzymatic pretreatments were tested, including hyaluronidase (Boehringer, Mannheim, Germany; 2 mg/ml in PBS pH 5 for 60 min at 37°C), pronase (Sigma, Deisenhofen, Germany; 2 mg/ml in PBS pH 7.3 for 60 min at 37°C), and bacterial protease XXIV (Sigma; 0.02 mg/ml; PBS pH 7.3 for 60 min at 37°C). Finally, the mouse monoclonal antibodies against Tob1 (Assay Designs, Ann Arbor, MI, USA) were used at a dilution of 1:20 without pretreatment of the sections.

### Amplification and cloning of *Tob1 *cDNA

RNA was isolated from differentiated ADTC5 cells (Ricken Library) in accordance with the extraction method with Trizol^® ^(Invitrogen) and reverse-transcribed into cDNA with SuperScript II™ reverse transcriptase (Invitrogen) by following the manufacturer's recommendation.

PCR amplification of a 607 base pair *Tob1 *cDNA fragment (nucleotides 402–1008 of the sequence in GenBank accession no. NM_009427) was performed with gene-specific primers (forward, 5'-GGAGCCCCCAGGTGTTCATGC-3'; reverse, 5'-CTCGTTGAGGCCTCCGTAGG-3') by a standard method, and amplification products were cloned into pCR^®^-BluntII-TOPO^® ^vector (Invitrogen).

### *In situ *hybridization

*In situ *hybridization of sectioned appendicular skeleton from newborn mice was performed with digoxigenin-labeled antisense riboprobes transcribed from the *Tob1 *cDNA fragment. Hindlegs of newborn mice were fixed overnight in 4% paraformaldehyde resolved in PBS. After stepwise transfer through solutions with increasing ethanol concentration, the specimens were incubated in xylene and finally embedded in paraffin wax.

For *in situ *hybridization, paraffin-embedded samples were cut into slices 7 μm thick and mounted on microscope slides. The sections were hybridized with digoxigenin-11-UTP-labeled antisense riboprobes, which were transcribed with T7 RNA polymerase from the *Tob1 *cDNA fragment cloned into pCR^®^-BluntII-TOPO^® ^(Invitrogen), after linearization of the plasmid with *Bam*HI.

*In situ *hybridization was performed as described by Dietz and colleagues [14]. After detection of hybridization products, the sections were mounted under coverslips in Kaiser's glycerol gelatin (Merck) and photographed under a Zeiss Axioplan 2 microscope.

## Results

### Construction of the SensiChip cartilage microarray

**Figure 1:**
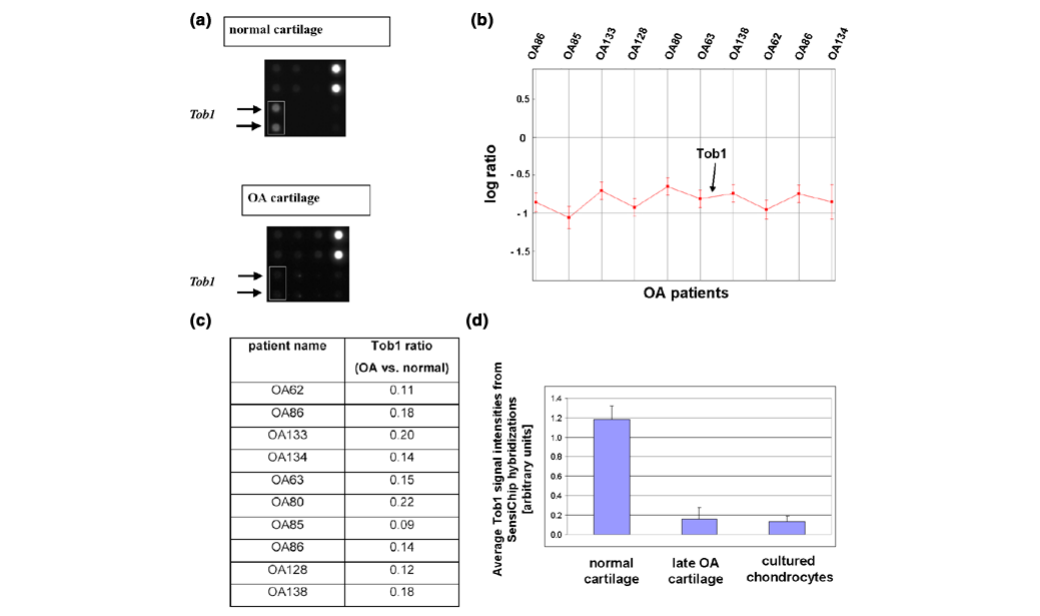
*Tob1 *expression in normal and osteoarthritic cartilage (oligonucleotide array experiments). **(a) **Area of one customized SensiChip microarray illustrating the 70-mer oligonucleotide spots that represent the 3'-untranslated region of human *Tob1 *and demonstrate its differential expression between normal and one late-stage osteoarthritic cartilage. **(b) **Trend plot demonstrating the transcriptional downregulation of human *Tob1 *in all RNA samples from cartilage of late-stage osteoarthritis patients used for SensiChip hybridization experiments. The logarithmic ratio of differential *Tob1 *expression calculated by the software Resolver is plotted against the corresponding osteoarthritic patient sample used for expression profiling. Error bars indicate standard deviations of ratios. *P *values for the ratios of all 10 osteoarthritic samples were less than 0.05. **(c) **Transcriptional downregulation of human *Tob1 *in late-stage osteoarthritic cartilage samples from 10 human donors. All *Tob1 *ratios were calculated by the gene expression analysis software Resolver. *P *values for the ratios of all 10 osteoarthritic samples were less than 0.05. **(d) **Plot of average *Tob1 *signal intensities from independent SensiChip microarray hybridizations using RNA samples from normal cartilage, late-stage osteoarthritic cartilage and cultured primary human chondrocytes. *Tob1 *signal intensities from independent SensiChip hybridizations of RNA samples from pooled normal cartilage (10 hybridization experiments), 10 late-stage osteoarthritis patients (10 hybridization experiments) and 5 different cell culture samples of proliferating primary human chondrocytes (5 hybridization experiments) were merged respectively and are plotted as average *Tob1 *signal intensities. Error bars indicate standard deviations of *Tob1 *signal intensities.

A microarray covering 340 human cartilage relevant genes was constructed, where one single gene was represented by one 70-mer oligonucleotide (Fig. 1a). Most genes were selected from the literature and have important roles in anabolic or catabolic pathways during osteoarthritis (for example, cartilage matrix proteins such as collagens, relevant degrading enzymes such as MMPs and aggrecanases, and genes from important catabolic [IL-1, tumor necrosis factor-α] and anabolic [BMP, transforming growth factor-β] signaling pathways).

### Gene expression analysis: differentially expressed genes

**Figure 2:**
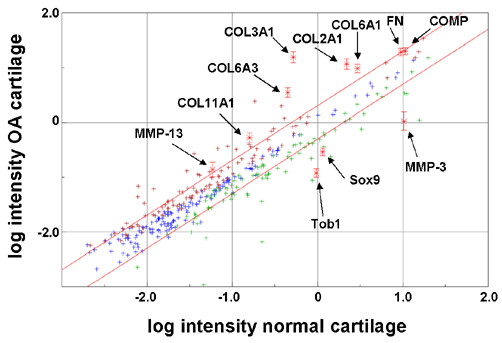
Plot of normalized logarithmic expression signal intensities RNAs from late osteoarthritic against the intensities for normal cartilage. RNA samples from 10 late-stage osteoarthritis patients were hybridized in comparison with normal cartilage (pool of nine donors) on SensiChip microarrays. After normalization, expression data were merged and corresponding signal intensities of late-stage osteoarthritis patients and normal cartilage were plotted against each other. Several differentially expressed marker genes (*P *< 0.01) are highlighted and diagonal lines indicate a twofold regulation. Error bars show standard deviations of ratios.

Total RNAs from 10 late-stage osteoarthritic cartilage samples were hybridized separately against a pool of mixed total RNAs from nine normal cartilage donors on the customized SensiChip microarrays. Merging of expression profiles obtained from all 10 late-stage osteoarthritic cartilage samples used for hybridizations resulted in about 200 significantly regulated genes that were differentially expressed between normal and osteoarthritic cartilage, with *P *< 0.01 (Fig. 2 and Table 2; the whole data set is in Additional file 1).

### *Tob1 *is repressed in osteoarthritic chondrocytes

One of the differentially expressed genes was the human transducer of ERBB2,1 (*Tob1*; GenBank accession no. NM_005749). *Tob1 *was transcriptionally downregulated in all 10 human osteoarthritic cartilage samples to, on average, one-sixth (Fig. 1). Corresponding *P *values were less than 0.05 for all human OA samples.

### Confirmation of *Tob1 *expression and regulation by (quantitative) PCR and immunostaining in normal and osteoarthritic articular cartilage

**Figure 3:**
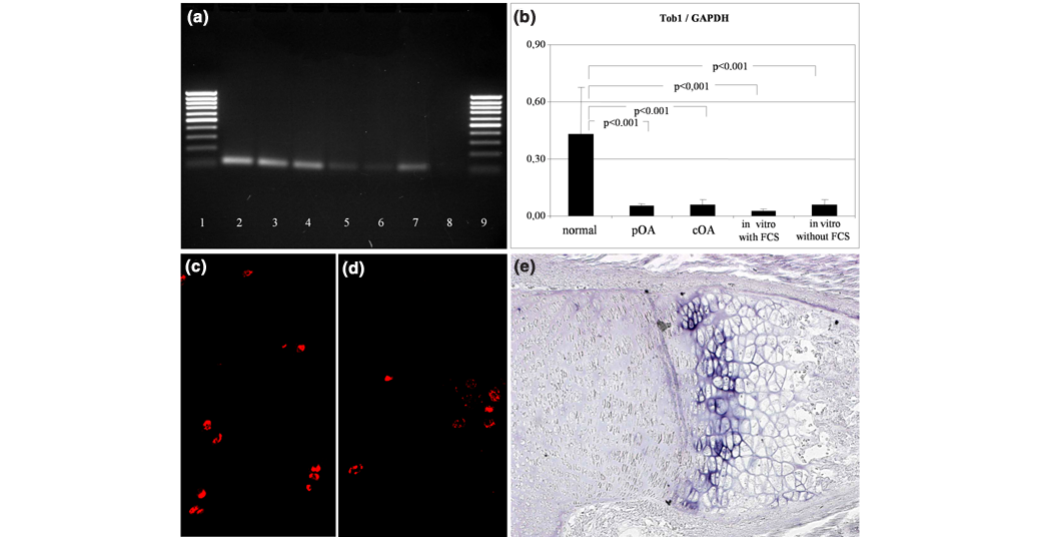
*Tob1 *expression in fetal, normal and osteoarthritic cartilage (PCR, immunostaining, *in situ *hybridization). **(a) **Conventional PCR demonstrates the expression of *Tob1 *in normal and (at a reduced level) in osteoarthritic cartilage samples (lanes 1 and 9, molecular weight standards; lanes 2–4, normal cartilages; lanes 5–7, osteoarthritic cartilages; lane 8, negative control). In all experiments the RNA was directly from the tissue (without isolation of cells before isolation of RNA). **(b) **Quantitative real-time PCR analysis for mRNA expression levels of *Tob1 *in normal (*n *= 10) and peripheral (pOA, *n *= 8) and central (cOA, *n *= 7) osteoarthritic cartilage as well as normal adult articular chondrocytes cultured with (*n *= 6) and without (*n *= 3) serum. Results are shown as ratios to glyceraldehyde-3-phosphate dehydrogenase. **(c, d) **Immunolocalization of Tob1 in human normal (c) and osteoarthritic (d) articular cartilage (in both the middle and upper deep zones of the cartilage are shown). **(e) **mRNA expression analysis of *Tob1 *in fetal growth plate cartilage of mice, with the use of *in situ *hybridization: detectable expression levels are restricted to the hypertrophic zone (and osteoblasts).

Conventional PCR confirmed the expression of *Tob1*, both in normal (*n *= 3) and osteoarthritic (*n *= 3) chondrocytes, with a weaker signal detected in the osteoarthritic samples (Fig. 3a). To validate and quantify differential regulation of *Tob1*, qPCR was performed on a set of normal (*n *= 10) and osteoarthritic (*n *= 15) samples. These experiments confirmed both its expression in normal articular cartilage and a highly significant decrease in *Tob1 *transcript levels in osteoarthritic samples (7.8-fold; *P *< 0.001; Fig. 3b).

Immunolocalization with monoclonal antibodies against Tob1 showed the presence of *Tob1 *protein in normal (*n *= 5) and osteoarthritic (*n *= 8) articular chondrocytes (Fig. 3c,d). A somewhat weaker staining was observed in the osteoarthritic specimens than in the normal specimens, but this was not quantifiable because of the immunostaining technology used.

### Correlation of *Tob1 *expression to markers for chondrocyte anabolism, catabolism, and proliferation

**Figure 4:**
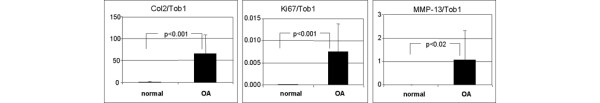
Comparative analysis of mRNA expression levels of collagen type II (*Col2*), *Ki-67*, and *MMP-13 *relative to *Tob1 *in normal and osteoarthritic chondrocytes.

Next we examined whether *Tob1 *gene expression levels were correlated with the expression of marker genes of cell proliferation (*Ki-67*) and anabolic (*collagen type II*) and catabolic (*MMP-13*) activation of articular chondrocytes. This analysis showed highly significant correlations between these genes in osteoarthritic compared with normal chondrocytes (Fig. 4).

### Expression of *Tob1 *in articular chondrocytes *in vitro*

*Tob1 *was expressed in isolated human adult articular chondrocytes *in vitro*. The mRNA expression levels of *Tob1 in vitro *were comparable to those of osteoarthritic chondrocytes *in situ *and were therefore significantly lower than those of normal chondrocytes *in situ *(oligo-array, Fig. 1d; qPCR, Fig. 3b). It is noteworthy that *Tob1 *was more strongly expressed in cells cultured without serum than with it. No significant regulation of *Tob1 *was found by two major anabolic (BMP-7) and catabolic (IL-1β) mediators in adult articular cartilage in cultured chondrocytes *in vitro *(data not shown).

### Expression of *Tob1 *in the fetal growth plate and during chondrocyte differentiation *in vitro*

*In situ *hybridization on mouse fetal growth plate cartilage was performed to assess differential expression in the different cartilage zones. This showed that the expression of *Tob1 *was concentrated in the hypertrophic zone (zone of terminal differentiation and cessation of proliferation). Cells of the resting and proliferating zones (that is, areas of proliferation and matrix synthesis) showed no or very much weaker staining (Fig. 3e). In addition, osteoblasts were positive (not shown).

**Figure 5:**
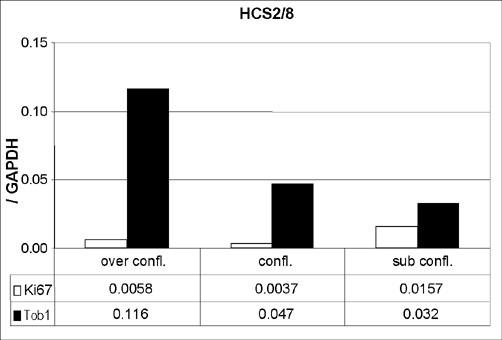
Comparative mRNA analysis for *Tob1 *and proliferation associated *Ki-67 *mRNA expression in chondrocytic HCS-2/8 cells. HCS-2/8 cells were cultured in sub-confluent, confluent, and over-confluent conditions and the *Tob1 *mRNA levels determined by qPCR (shown are the ratios to glyceraldehyde-3-phosphate dehydrogenase (GADPH)).

Expression profiling in HCS-2/8 cells, which are known to show a more differentiated phenotype in high-density cultures than when cultured in subconfluent or confluent status [15] showed an inverse relationship between *Tob1 *expression and the proliferation marker *Ki-67 *(Fig. 5).

## Discussion

Differential gene expression analysis, as performed by us on normal and osteoarthritic chondrocytes, reveals long lists of differentially expressed genes of potential interest for furthering the understanding, diagnosis and/or modulation of osteoarthritis. The genes identified might be interesting with regard to any of these three aspects, but careful validation is needed to confirm the relevance of the findings obtained. In this regard, three levels of validation have to be achieved: (1) technical validation of screening results, (2) functional validation of the gene *in situ *or *in vitro*, and finally (3) establishment of relevance of the gene for the (physiology and/or) pathophysiology of the tissue.

In our oligonucleotide-based array screen we detected many known differentially expressed genes. Thus, many marker genes behaved as expected from previous investigations: stromelysin I (*MMP-3*) [7] and the cartilage transcription factor *SOX9 *[16] were significantly downregulated, whereas many constituents of the extracellular matrix were significantly upregulated (*collagen types II *[17], *III *[18], *VI *[19], *COMP *[20], and *fibronectin *[21]). Further, MMP-13, the major collagenase of osteoarthritic cartilage [22,23], was induced [7]). Taken together, these findings validated this gene array technology as a reliable tool for identifying differentially expressed genes. In addition, many genes previously unknown to be differentially regulated in osteoarthritic cartilage were detected.

Among the new differentially expressed genes we identified *Tob1 *as being significantly downregulated in osteoarthritic compared with normal articular chondrocytes. For technical validation (validation level I), this was confirmed by conventional and quantitative PCR at a very high significance level. Immunostaining provided additional evidence of the presence of Tob1 in normal and osteoarthritic chondrocytes.

*Tob1*, originally identified as binding partner of Erb ('transducer of Erb' [24]), is a member of a larger family of proteins, which share common protein domains and are known to exert anti-proliferative and phenotype-stabilizing effects on various cell types including osteoblasts ([24,25]; reviewed in [4] and [5]).

Thus, to obtain insights into the functional activity of *Tob1 *in articular cartilage (validation level II), we correlated Tob1 expression with the expression of the *Ki-67 *antigen, a well-established gene expressed only by cells in the proliferation phase [26]. We found a highly significant inverse correlation between *Tob1 *expression and proliferative activity of chondrocytes. It is noteworthy that after isolation from the articular matrix *Tob1 *was also repressed in normal articular chondrocytes *in vitro*. This might well reflect the fact that adult articular chondrocytes show an increased proliferative activity and also enhanced anabolic [27] and catabolic activity [7] after removal from the tissue. The fact that cells cultured with serum *in vitro *showed even lower *Tob1 *expression levels than cultures without serum further supports this notion, because serum is known to increase proliferation of chondrocytes *in vitro *[28]. In addition, the chondrocytic cell line HCS-2/8 showed an inverse relationship between proliferative activity and cell differentiation on the one hand and Tob1 expression on the other. Interestingly, fetal chondrocytes *in situ *selectively express Tob1 in the hypertrophic zone, which is in contrast to other zones where no proliferative activity is seen [25]. This indicates that *Tob1 *expression in chondrocytes is inversely related to proliferation in a similar way to that seen in T cells [29]. Another basic effect of *Tob1 *is also observed in chondrocytes: a repression of *Tob1 *is needed before activation of otherwise quiescent T cells [29,30]; similarly, there is a clearcut inverse correlation between (anabolic and catabolic) chondrocyte activity and Tob1 expression.

In many respects the downregulation of *Tob1 *fits well into the scenarios taking place during osteoarthritis (validation level III), which suggests that *Tob1 *is a potential key molecule of cell phenotype regulation in osteoarthritic chondrocytes. Thus, in osteoarthritic cartilage an increase in proliferation [31-35] is found, whereas hardly any proliferative activity exists in normal articular adult cartilage [31,32]. These cells seem to be G0-arrested, quiescent and phenotypically stable, in other words exactly the cell type that would be expected to express high levels of *Tob1 *[4,29]. It is noteworthy that both phenotypic instability [36] and anabolic activation [17] are key features of osteoarthritic chondrocytes, fitting well to the downregulation of *Tob1*.

*Tob1 *seems in many circumstances and, in particular, in skeletal cells to interact with the BMP pathway [37]. Tob1-knockout mice develop osteopetrosis due to a lack of inhibition of BMP-stimulated bone growth [37]. In addition, overexpression of *Tob1 *reduces BMP2 signaling [38]. Although in *Tob1*-knockout mice no specific 'hyperplastic' cartilage phenotype was obvious, BMP-2 and BMP-7 are reported to have important functions in cartilage homeostasis [39,40]. The presence of Tob1 could therefore explain why, despite the presence of BMPs within articular cartilage [39], normal chondrocytes show only very low anabolic activity. By the same argument, osteoarthritic chondrocytes BMPs might have much more anabolic potential, a feature recently suggested in studies *in vitro *[27].

In sum, our study provides for the first time compelling evidence of the expression and presence of Tob1 as a new intracellular mediator in adult articular chondrocytes and its downregulation in the osteoarthritic disease process. Tob1 fits well functionally with the cellular biological changes found in this condition such as proliferation, activation and the loss of a differentiated phenotype. Our data, together with the knowledge from other cellular systems in the literature, suggest that *Tob1 *is a key molecule in the scenario of cellular alterations of osteoarthritis.

## Conclusions

Oligonucleotide-based microarray analysis was used to screen for differences in gene expression levels in between normal and osteoarthritic chondrocytes. Among other genes, *Tob1 *was identified as being significantly downregulated in osteoarthritic chondrocytes. Correlative gene expression studies on cellular features such as cell proliferation, cell activation and the loss of a differentiated phenotype suggest that downregulation of *Tob1 *expression might be an important aspect of cellular processes in osteoarthritic cartilage degeneration.

## Abbreviations

BMP = bone morphogenetic protein; cDNA = complementary DNA; cRNA = complementary RNA; IL = interleukin; MMP = matrix metalloproteinase; PBS = phosphate-buffered saline; PCR = polymerase chain reaction; qPCR = quantitative polymerase chain reaction; UTR = untranslated region.

## Competing interests

The author(s) declare that they have no competing interests. MG, JS, UD, and EB are all employed by Sanofi-Aventis as research scientists. The publication is a result of a scientific collaboration between industry and the other academic authors. The protein Tob1 is not pursued as a project within the osteoarthritis portfolio of Sanofi-Aventis; therefore the industry-affiliated authors have stated that they and the company have no competing interests.

## Authors' contributions

MG performed the gene expression analysis. JS cultured the HCS-2/8 cells and contributed to the bioinformatic analysis of obtained data sets. JH performed the collection and processing of human material (including RNA isolation). UD performed the *in situ *hybridization analysis. MT contributed the HCS-2/8 cell line. EB participated in the design of the study and coordinated the gene expression experiments including the bioinformatic analysis. TA wrote most of the manuscript and participated in the design of the study. His group contributed the TaqMan, conventional PCR and immunohistochemical analyses (together with JH). All authors contributed to writing and correcting the manuscript and have approved the final version.

## Supplementary Material

Additional File 1 An Excel file that contains details of 200 significantly regulated genes that were differentially expressed between normal and osteoarthritic cartilage with *P *values <0.01. Click here for file

## Figures and Tables

**Table 1:** Sequences of primers and probes for quantitative real-time polymerase chain reaction

Gene	GenBank accession no.	Primers (5'→3')	Conc. (nM)	Probe (5'→3')	MgCl_2 _(mM)
*GAPDH*	NM_002046	Forward: GAAGGTGAAGGTCGGAGTC	50	CAAGCTTCCCGTTCTCAGCC	5.5
		Reverse: GAAGATGGTGATGGGATTTC	900		
*TOB1*	NM_005749	Forward: TCTGCTGCTGTAAGCCCTACCT	300	CGGTCCACTCAGCCTTTAACCTTTACCACT	6.5
		Reverse: TTCATTTTGGTAGAGCCGAACTT	900		
*Ki-67*	NM_002417	Forward: CAGTGATCAACGCCGTAGGTC	900	CTTCCAGCAGCAAATCTCAGACAGAGGTTC	6.0
		Reverse: TCGGCTGATAGACACTCTCTTTTG	900		
*COL2A1*	NM_001844	Forward:CAACACTGCCAACGTCCAGAT	50	ACCTTCCTACGCCTGCTGTCCACG	5.5
		Reverse: CTGCTTCGTCCAGATAGGCAAT	300		
*MMP-13*	NM_002427	Forward: TCCTCTTCTTGAGCTGGACTCATT	900	TCCTCAGACAAATCATCTTCATCACCACCAC	7.0
		Reverse: CGCTCTGCAAACTGGAGGTC	50		

*GAPDH*, glyceraldehyde-3-phosphate dehydrogenase; *MMP*, matrix metalloproteinase; *COL2A1*, collagen type II (alpha 1 chain); *TOB1*, Transducer of ERBB2.

**Table 2:** Table showing genes which were upregulated or downregulated in osteoarthritic chondrocytes (changes in mRNA expression levels >2-fold; *P *< 0.01)

Downregulated genes	GenBank accession no.	OA:N	OA	N	Upregulated genes	GenBank accession no.	OA:N	OA	N
*CHI3L1*	NM_001276	0.02	0.001	0.05	*COMP*	NM_000095	2.07	19.93	10.68
*Follistatin*	NM_006350	0.06	0.01	0.22	*BGLAP*	X53698	2.18	0.13	0.07
*APOD*	NM_001647	0.08	1.10	15.68	*TIMP1*	X03124	2.20	12.18	6.34
*EDR2*	NM_004427	0.09	0.09	0.86	*ARGBP2*	AF049884	2.25	0.11	0.04
*MMP3*	X05232	0.09	1.04	10.28	*FGF18*	AF075292	2.35	0.40	0.16
*Tob1*	NM_005749	0.14	0.12	0.96	*Fibronectin*	M10905	2.36	19.08	9.46
*SLC3A2*	NM_002394	0.19	0.04	0.22	*Frizzled homolog 1*	NM_003505	2.37	0.05	0.03
*MAPK7*	NM_002749	0.22	0.01	0.05	*Choriolysin h 2*	AA829685	2.38	0.06	0.03
*SOX9*	Z46629	0.26	0.29	1.14	*CRTL1*	NM_001884	2.43	0.76	0.31
*SERPING1*	NM_000062	0.27	0.76	3.27	*MKP-L*	NM_007026	2.57	0.32	0.11
*TGFBR3*	NM_003243	0.27	0.05	0.15	*THBS3*	NM_007112	2.59	0.11	0.04
*FRZB*	NM_001463	0.27	0.23	1.43	*ADAMTS2*	NM_014244	2.68	0.06	0.02
*MRS3/4*	AF327402	0.28	0.12	0.43	*ADAMTS1*	NM_006988	2.70	0.12	0.05
*UBC*	M26880	0.31	3.29	11.04	*CHM-I*	NM_007015	2.88	0.36	0.09
*Integrin α5*	NM_002205	0.35	0.36	1.17	*MMP-13*	X75308	2.88	0.13	0.06
*GNB2L1*	NM_006098	0.38	1.15	2.39	*COL6A1*	X15880	2.89	9.55	2.94
*NF-κB_p65*	Q04206	0.40	0.04	0.10	*COL11A1*	J04177	3.84	0.52	0.16
*Pim-1*	Z58595	0.41	0.05	0.15	*TNFAIP6*	NM_007115	4.73	0.33	0.07
*NCK1*	NM_006153	0.41	0.01	0.04	*Thrombospondin 2*	NM_003247	5.01	0.13	0.03
*GSS*	NM_000178	0.43	0.03	0.06	*COL2A1*	X16468	5.67	11.62	2.20
*HLA-B*	M81798	0.43	1.43	3.50	*SPP1*	NM_000582	5.78	1.96	0.38
*ICAM1*	NM_000201	0.44	0.09	0.26	*CKTSF1B1*	NM_013372	5.89	0.06	0.01
*TG-interacting factor*	NM_003244	0.47	0.05	0.10	*COL6A3*	NM_004369	8.70	3.49	0.45
*Phosphomannomutase 1*	U86070	0.47	0.11	0.26	*COL1A2*	X55525	14.25	4.21	0.33
*DLX5*	NM_005221	0.47	0.04	0.08	*TGFBI*	NM_000358	14.67	2.41	0.18
*Biglycan*	NM_001711	0.49	0.21	0.50	*COL3A1*	X14420	31.33	15.33	0.52
TP53	NM_000546	0.50	0.14	0.34					

N, mean of mRNA expression levels in the normal cartilage samples (in arbitrary units); OA, mean of mRNA expression levels in the osteoarthritic cartilage samples (in arbitrary units); OA:N, ratio of osteoarthritic to normal.
